# High Abundance of Intratumoral γδ T Cells Favors a Better Prognosis in Head and Neck Squamous Cell Carcinoma: A Bioinformatic Analysis

**DOI:** 10.3389/fimmu.2020.573920

**Published:** 2020-09-30

**Authors:** Huanzi Lu, Wenxiao Dai, Junyi Guo, Dikan Wang, Shuqiong Wen, Lisa Yang, Dongjia Lin, Wenqiang Xie, Liling Wen, Juan Fang, Zhi Wang

**Affiliations:** Hospital of Stomatology, Guanghua School of Stomatology, Guangdong Provincial Key Laboratory of Stomatology, Sun Yat-sen University, Guangzhou, China

**Keywords:** head and neck squamous cell carcinoma (HNSCC), γδ T cells, butyrophilin, BTN3A1, MICB, TCGA, ImmuCellAI, CIBERSORTx

## Abstract

γδ T cells are a small subset of unconventional T cells that are enriched in the mucosal areas, and are responsible for pathogen clearance and maintaining integrity. However, the role of γδ T cells in head and neck squamous cell carcinoma (HNSCC) is largely unknown. Here, by using RNA-seq data from The Cancer Genome Atlas (TCGA), we discovered that HNSCC patients with higher levels of γδ T cells were positively associated with lower clinical stages and better overall survival, and high abundance of γδ T cells was positively correlated with CD8+/CD4+ T cell infiltration. Gene ontology and pathway analyses showed that genes associated with T cell activation, proliferation, effector functions, cytotoxicity, and chemokine production were enriched in the group with a higher γδ T cell abundance. Furthermore, we found that the abundance of γδ T cells was positively associated with the expression of the butyrophilin (BTN) family proteins BTN3A1/BTN3A2/BTN3A3 and BTN2A1, but only MICB, one of the ligands of NKG2D, was involved in the activation of γδ T cells, indicating that the BTN family proteins might be involved in the activation and proliferation of γδ T cells in the tumor microenvironment of HNSCC. Our results indicated that γδ T cells, along with their ligands, are promising targets in HNSCC with great prognostic values and treatment potentials.

## Introduction

Head and neck cancer is a group of cancers that occurs in the head and neck region, including the oral cavity, pharynx and larynx, of which more than 90% cases are squamous cell carcinoma (1). Head and neck squamous cell carcinoma (HNSCC) is the seventh most common cancer globally in 2018, accounting for 3% of all cases of cancer incidence and 1.5% of all cancer deaths annually (2). Although the treatment of HNSCC has evolved from surgery to a multidisciplinary treatment including surgery, radiotherapy, chemotherapy and molecular targeted therapy in the past few decades, the 5-year overall survival (OS) of HNSCC patients has not been significantly improved, remaining at ~60% (3). In addition, more than 50% of HNSCC patients have a locoregional recurrence or distant metastasis within 3 years, which results in a poorer prognosis (4). In recent years, with the advancement of cancer immunotherapy, some patients with advanced cancer have benefited from it, but the response rate in HNSCC patients is only ~13–20% (4, 5). Therefore, it is urgent to find novel molecular markers or cell types with therapeutic and prognostic values in HNSCC.

T cells are a group of immune cells that play the key role in antitumor immune response. Based on the composition of T cell receptors (TCR), T cells are mainly divided into αβ T cells and γδ T cells (6). αβ T cells, including CD4+ T cells and CD8+ T cells, have been extensively studied in tumor immunity, but the role of γδ T cells in the tumor microenvironment (TME) is largely unknown. γδ T cells account for only 1–5% of total T cells in the peripheral blood, but they mainly reside in mucosal areas, such as the intestinal epithelia and oral mucosa, accounting for 10–100% of intraepithelial lymphocytes (7). Generally, γδ T cells are responsible for pathogen clearance and maintaining integrity of the epithelium (7). According to the arrangement of the Vδ chain, human γδ T cells are classified into three different subsets, namely Vδ1 T cells, Vδ2 T cells, and Vδ3 T cells which are only found in liver (8). Vδ2 T cells are the main subset of circulating γδ T cells in the peripheral blood, accounting for about 50 ~ 90% γδ T cells (9).In addition, Vδ2 T cells also accumulate in tumor tissues and exert potent cytotoxic activity, indicating that these cells possess a potential antitumor activity. In contrast, Vδ1 T cells are mainly distributed in mucosal areas, playing an important role in killing bacteria or viruses and maintaining tissue homeostasis, in addition, they are also involved in antitumor immunity (10).

Recent studies have reported that the infiltration level of cytotoxic T cells or NK cells in the TME is usually correlated with an improved prognosis in various kinds of cancers (11). γδ T cells, as one of the cytotoxic T cells that infiltrates tumor tissue, are reported to possess a high cytotoxic activity in lung cancer, breast cancer, colon cancer and gastric cancer, and are related to the improvement of overall survival (12). However, recent studies showed that γδ T cells may also promote tumor progression (13). However, until now, the functions and prognostic value of γδ T cells in HNSCC have been rarely studied. A previous study explored the γδ T cells in the peripheral blood of HNSCC patients and found that there was no correlation between γδ T cell abundance and T stages (14), but the abundance of γδ T cells in the TME has not been studied.

Unlike conventional T cells, which recognize antigens presented by tumor cells or antigen presenting cells (APC) through major histocompatibility complex (MHC) molecules, γδ T cells recognize various types of antigens without MHC restriction (15), but the exact mechanisms that trigger γδ T cell activation and proliferation are largely unknown. Notably, recent studies have found that Vδ2 T cells are activated by phosphoantigens (P-Ags) produced by malignant cells or infected cells through the presentation of butyrophilin family proteins (BTNs) to TCRγδ on Vδ2 T cells (16). BTNs belong to the type I transmembrane proteins of the immunoglobulin superfamily. In humans, these proteins can be classified into BTN1, BTN2, and BTN3 subfamilies, including BTN1A1, BTN2A1-2, and BTN3A1-3 (17). Among them, BTN2A1 and BTN3A1-3 have been reported to take up and present P-Ags to the TCR of γδ T cells (18–20). Studies have shown that through the binding to P-Ags presented by BTN3A1 and BTN2A1 on infected or malignant cells, γδ T cells could be activated, proliferate rapidly, and release cytokines to induce anti-infection or antitumor responses (20, 21). Recent studies have found that a higher expression of BTN3A2 in ovarian cancer and triple negative breast cancer is positively correlated with increased T cell infiltration and a better prognosis (22, 23), but other studies have reported that BTN3A2 is associated with poor prognosis in gastric cancer and pancreatic ductal adenocarcinoma (PDAC) (22, 24). In addition, the engagement of natural killer group 2 member D (NKG2D) with its ligands provides the costimulatory signaling pathway that activates γδ T cells (25). The NKG2D ligands (NKG2DLs) include MHC class I polypeptide-related sequence A and B (MICA/MICB) and the UL16 binding protein 1-6 (ULBP1-6) (26). However, the potential correlation of γδ T cells and BTN families in the TME of HNSCC is still unclear, and whether the NKG2DL-NKG2D pathway participates in the antitumor immunity of HNSCC remains to be discovered.

In this study, by using the patient cohorts from TCGA database, we discovered that HNSCC patients with a higher abundance of γδ T cells had prolonged overall survival, suggesting that γδ T cells were of great prognostic values for HNSCC, and were highly correlated with CD8+ T cell and CD4+ T cell infiltration in the TME. We further found that activation of γδ T cells in the TME was associated with the BTN family proteins. This study revealed the prognostic value of γδ T cells in head and neck cancer, and revealed the possible activation mechanisms of γδ T cells in HNSCC.

## Materials and Methods

### HNSCC and CESC Datasets From TCGA Database

Data on the HNSCC patient cohort was downloaded from The Cancer Genome Atlas (TCGA). Patients without RNA-seq data were excluded. A total of 537 samples were included in this study, including 494 tumor tissue samples and 43 adjacent normal tissue samples, all of the 43 adjacent normal tissue samples were classified into normal group. The phenotype information and survival data of HNSCC patients (version: 08-07-2019) and RNA-seq data (version: 07-19-2019) were downloaded from UCSC Xena (https://xenabrowser.net/datapages/). In addition, the RNA-seq data (version: 07-19-2019), phenotype (version: 08-07-2019) data and survival data (version: 07-19-2019) of a cervical squamous cell carcinoma (CESC) patient cohort were also downloaded from UCSC Xena. Patients without RNA-seq data were excluded. A total of 281 samples were included in this study, including 278 tumor tissue samples and 3 adjacent normal tissue samples. The classification of T stage, N stage and clinical stage of each patient was based on American Joint Committee of Cancer (AJCC, 7th edition) (27). The RNA-seq data included Fragments per Kilobase Million (FPKM) matrix and a counts matrix and to reduce biases among different samples, the FPKM format was converted into a Transcripts Per Million (TPM) format for further analysis (28). The function for the conversion of FPKM to TPM is listed below, and this process was calculated by R language:

TPM(i) = (FPKM(i)/sum(FPKM all transcripts)) × 10^6^ (where i refers to the specific gene of specific sample in the expression matrix).

After conversion, the sum of all transcripts in each sample was on the order of 10^6^, which makes it more convincing to compare the expression levels of specific genes across different samples.

### Gene Expression Comparison and Correlation Analysis

γδ T cell abundance is defined by the geometric mean of the TPM values of TRDC/TRGC1/TRGC2, where TRDC encodes the constant chain of TCRδ, and TRGC1 and TRGC2 encode the constant chain of TCRγ. Based on the median expression of the geometric mean, the HNSCC patients were dichotomized into γδT-hi and γδT-lo groups (247 patients in each group, Table 1). Furthermore, to explore the effects of the BTN family proteins on the prognosis of HNSCC patients, the cohort was dichotomize based on the median expression levels of BTN3A1/BTN3A2/BTN3A3/BTN2A1, respectively. The effector and cytotoxic functions of γδ T cells were assessed by the expression levels of IFNG (interferon-γ), GZMA (granzyme A), GZMB (granzyme B), and GNLY (granulysin). The activation degree of cytotoxic γδ T cells is reflected by transcription factor Hobit (encoded by ZNF683) and activation receptor NKG2D (encoded by KLRK1). The difference in the ability to recruit CD8+ T cells between the γδT-hi and γδT-lo groups was shown by the expression levels of the chemokines CCL5 and CXCL9, which was reported previously (29). The expression levels of γδ T cell activation ligands among the γδT-hi, γδT-lo and normal groups are shown by the expression levels of ligands that can be recognized and bound by TCRγδ (BTN2A1, BTN3A1-3), and ligands that bind to NKG2D and activate γδ T cells (MICA, MICB, and ULBP1-6). Boxplots, heatmap and scatter plots were drawn by using R packages ggplot2, ggpubr and pheatmap. In the boxplots, the center line of box represents the median value, the upper and lower edges of box indicate the 75th and 25th percentiles, respectively. The whiskers extend 1.5 times the interquartile range (IQR) beyond the 75th and 25th percentiles of the box, respectively. Student's *t-*test was used to compare the difference in gene expression values between the two groups. Linear regression analysis was used to compare the correlation between each ligand and the geometric mean of the γδ T cell markers. For the linear regression analysis, the gene expression levels were first converted to log_10_ (TPM+1), and Pearson correlation coefficient *R*-values and *P*-values were obtained. Among these, the absolute value of R (|R|) > 0.7 was considered as a strong correlation, 0.4 < |R| <0.7 was considered as a moderate correlation, and |R| <0.4 was considered as a weak correlation. *P* < 0.05 is considered to be statistically significant.

**Table 1:** Baseline and clinical information of HNSCC patients in TCGA database.

**Factor**		**γδT-hi**	**γδT-lo**	***P-*value**
		**number(%)**	**number(%)**	
γδT cell marker expression (TPM)		0.60–19.03	0.00–0.59	
Gender	Male	179 (36.2%)	183 (37.0%)	0.684
	female	68 (13.8%)	64 (13.0%)	
Age	≥60y	148 (30.0%)	130 (26.3%)	0.103
	<60y	99 (20.0%)	117 (23.7%)	
Smoking history	Yes	154 (31.2%)	149 (30.2%)	0.644
	No	93 (18.8%)	98 (19.8%)	
Alcohol history	Yes	167 (33.8%)	160 (32.4%)	0.506
	No	80 (16.2%)	87 (17.6%)	
Tumor site	Oral cavity	140 (28.3%)	165 (33.4%)	0.003^**^
	Pharynx	53 (10.7%)	26 (5.3%)	
	Larynx	54 (10.9%)	56 (11.3%)	
T stage	1	25 (5.1%)	10 (2.0%)	0.016^*^
	2	80 (16.2%)	67 (13.6%)	
	3	59 (11.9%)	75 (15.2%)	
	4	83 (16.8%)	95 (19.2%)	
N stage	0	115 (23.5%)	129 (26.3%)	0.211
	1	38 (7.8%)	47 (9.6%)	
	2	88 (18.0%)	68 (13.9%)	
	3	3 (0.6%)	2 (0.4%)	
Clinical stage	1	15 (3.0%)	5 (1.0%)	0.020^*^
	2	46 (9.3%)	49 (9.9%)	
	3	44 (8.9%)	65 (13.2%)	
	4	142 (28.7%)	128 (25.9%)	
Perineural invasion	Yes	71 (14.4%)	93 (18.8%)	0.109
	No	98 (19.8%)	87 (17.6%)	
	Unknown	78 (15.8%)	67 (13.6%)	
HPV status	Yes	25 (5.1%)	5 (1.0%)	0.001^**^
	No	33 (6.7%)	39 (7.9%)	
	Unknown	189 (38.3%)	203 (41.1%)	

* P < 0.05,

** *P < 0.01*.

### Estimation of the Relative Abundance of Immune Cells in the RNA-seq Data

The relative abundance of immune cells in the RNA-seq data of HNSCC and CESC patients was estimated by using the newly developed online tool ImmuCellAI (Immune Cell Abundance Identifier), which is comprised of 24 immune cells, including 18 T cell subpopulations and another 6 immune cells (30). The relative abundances of 24 immune cells in tumors or normal tissues of HNSCC or CESC were downloaded from http://bioinfo.life.hust.edu.cn/ImmuCellAI#!/resource. In addition, the relative proportions of immune cells in the tumor or normal tissues of HNSCC were also calculated by using another online immune cell analytical tool, CIBERSORTx, which supports a deconvolution algorithm to evaluate the relative proportion of immune cells in tumor tissues (31). The composition of immune cells was calculated online (https://cibersortx.stanford.edu/).

### Analysis of Differentially Expressed Genes

The differentially expressed genes (DEG) in tumor tissues between the γδT-hi and γδT-lo groups were analyzed by R package DESeq2, and the selection criteria for DEGs were an absolute value of log2FoldChange > 1 and adjusted *P*-value (Padj) <0.05. Biological process (BP) in Gene Ontology (GO) and Kyoto Gene and Genome Encyclopedia (KEGG) term enrichment analyses were performed by R package clusterProfiler (version: 3.16.1) using the DEGs in the γδT-hi and γδT-lo group, respectively, the significance levels of enrichment results (adjusted *P*-values) were calculated by hypergeometric distribution. The Gene Set Enrichment Analysis (GSEA) was performed using the REACTOME database in MSigDB (version 7.1), and *P*-values for GSEA analyses were calculated by permutation tests. The normalized enrichment score (NES) >1, *P* < 0.05, and False Discovery Rates (FDR) <0.25 were considered statistically significant in GSEA analyses.

### Statistical Analyses

SPSS software version 25.0 was used for statistical analysis. The clinical parameters in this study included gender, age, smoking history, drinking history, tumor location, T stage, N stage, clinical stage, perineural invasion (PNI) and human papillomavirus (HPV) status. A Chi-square test was used to compare the clinical parameters between the two groups. OS was calculated and described by the Kaplan–Meier method. The difference of survival curves was tested by log-rank test. Univariate Cox proportional models were used to analyze the associations between clinical parameters and OS, and the factors with statistical significance were further included into multivariate Cox regression analysis. *P* < 0.05 was considered to be statistically significant (Wald test). GraphPad Prism version 7.0 was used to draw stacked histograms and survival curves of γδT-hi and γδT-lo groups. ns, not significant; ^*^P < 0.05; ^**^P < 0.01; ^***^P < 0.001; ^****^P < 0.0001.

## Results

### A High Abundance of γδ T Cells Is Significantly Correlated With Improved Survival of HNSCC Patients

**Figure 1:**
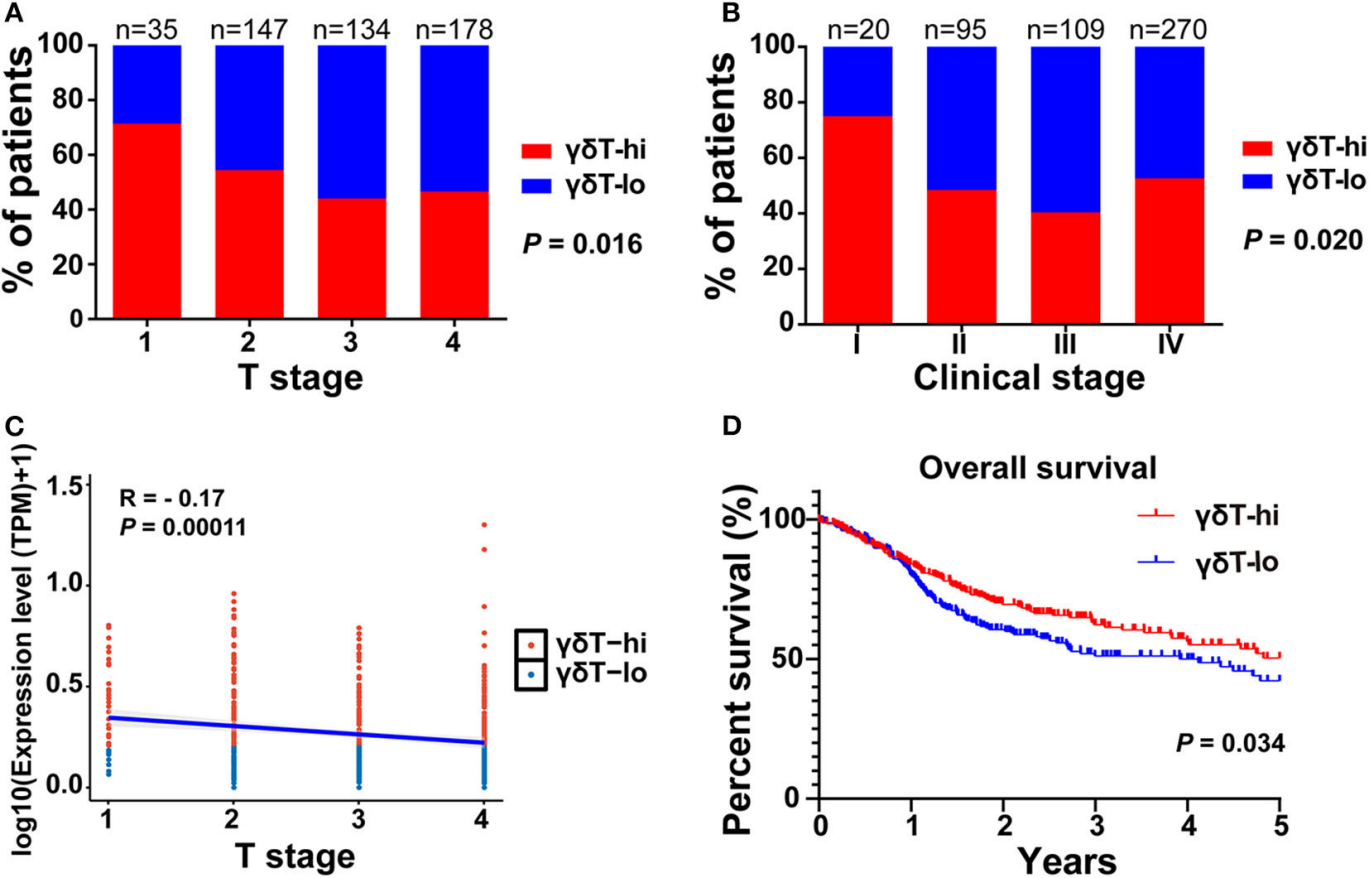
The distribution of T stages, clinical stages, and overall survival curves between the γδT-hi (*n* = 247) and γδT-lo (*n* = 247) groups in the HNSCC dataset. **(A)** Proportions of patients in each T stage (1–4) in the γδT-hi and γδT-lo groups. **(B)** Proportions of patients in each clinical stage (I-IV) in the γδT-hi and γδT-lo groups, the *P*-values in **(A,B)** were obtained by Chi-square test. **(C)** Correlation between T stages and γδ T cell markers in HNSCC, each point represents the tumor sample in each patient, the expression levels of γδ T cell markers were calculated by the geometric mean of TPM values of TRGC1, TRGC2 and TRDC, and were converted by log_10_ (TPM + 1). **(D)** Five-year overall survival curves in the γδT-hi and γδT-lo groups.

First, we compared the clinical parameters and the OS of HNSCC patients between the γδT-hi and γδT-lo groups. The results showed that patients in the γδT-hi group accounted for a higher proportion in the T1/T2 stages (Figure 1A, Table 1) and in phase I/II clinical stages (Figure 1B, Table 1), whereas patients in the γδT-lo group were more aggregated in the T3/T4 stages and phase III/IV stages. In addition, there was a negative correlation between T stages and γδT cell marker expression levels (*R* = −0.17, *P* < 0.05, Figure 1C). The overall survival curve of the two groups showed that the survival time of the γδT-hi group was significantly prolonged (*P* < 0.05, Figure 1D). Except for the tumor site and HPV status, the clinical parameters showed no significant differences between the two groups (Table 1). However, when these factors were analyzed for univariate analysis, only γδ T cell abundance, gender and PNI were significantly associated with the OS (Table 2). Furthermore, multivariate Cox regression analysis showed that only γδ T cell abundance and gender were statistically significant between the two groups (Table 2). These results showed that the abundance of γδ T cells was significantly correlated with the lower clinical stages and prolonged survival of HNSCC patients. To validate whether our findings could be replicated to other types of cancer, we studied an additional squamous cell carcinoma, CESC. The results showed that similar to our findings in HNSCC, CESC patients with a higher γδ T cell infiltration were positively correlated with a better OS (Supplementary Figure 1A) and the proportions of the γδT-hi group decreased in T3/4 stages, although there was no statistical significance among the T stages (*P* > 0.05, Supplementary Figure 1B).

**Table 2:** Univariate and multivariate survival analysis of HNSCC patients in TCGA database.

**Factor**	***P*-value**	**Hazard ratio**	***P*-value**
	**(Univariate)**	**(Multivariate)**	**(Multivariate)**
γδ T cell abundance	0.047^*^	0.734	0.036^*^
gender	0.023^*^	0.702	0.023^*^
age	0.295		
Smoking history	0.906		
Alcohol history	1.000		
tumor site	0.153		
T stage	0.297		
N stage	0.360		
Clinical stage	0.962		
Perineural invasion	<0.001^*^	1.066	0.058
HPV status	0.212		

* *P < 0.05*.

### γδ T Cell Abundance Is Positively Correlated With CD4+ and CD8+ T Cell Abundance in the HNSCC Samples

**Figure 2:**
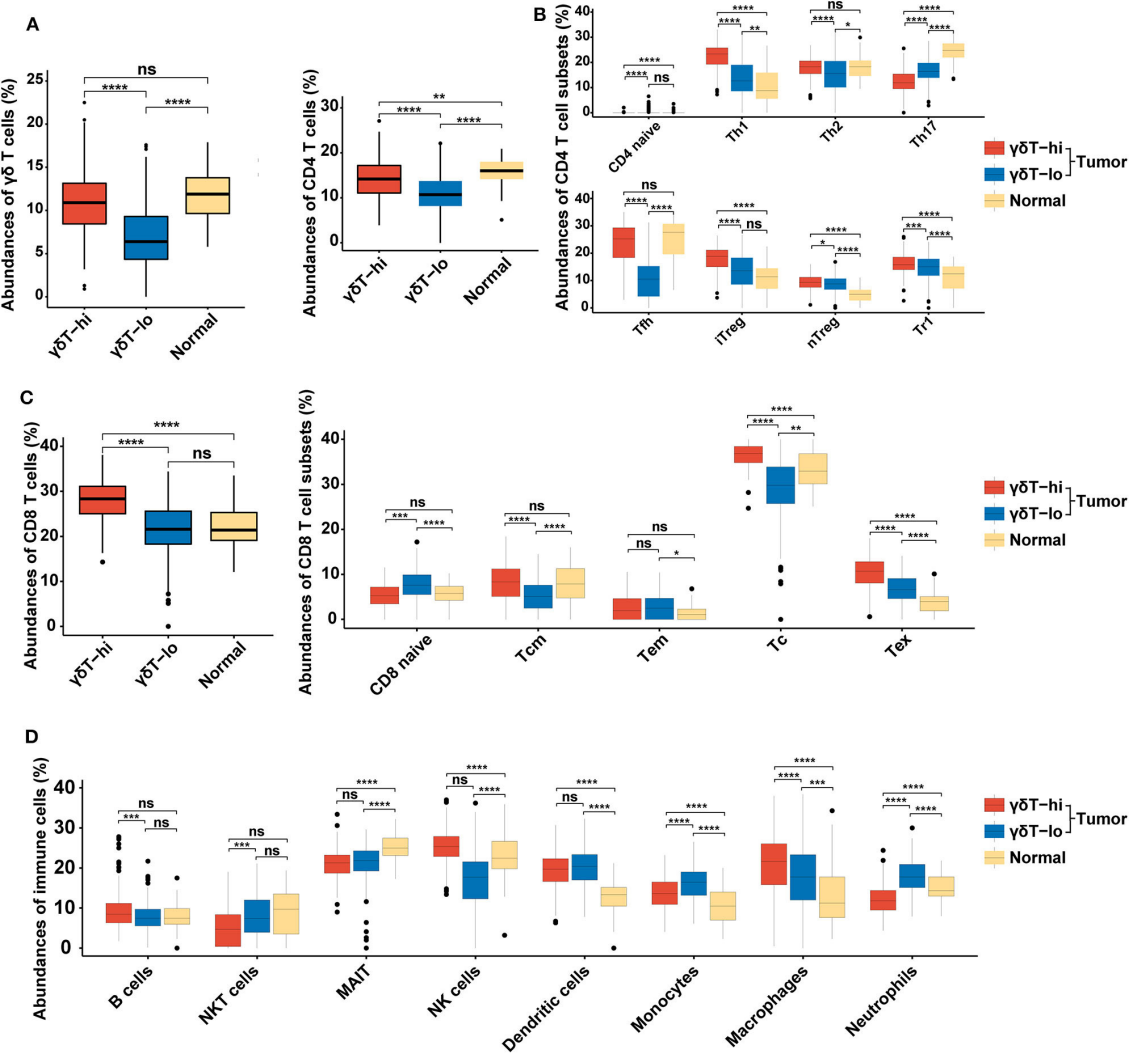
Boxplots showing the relative abundances of 24 cell types among the γδT-hi, γδT-lo and normal groups in HNSCC dataset using ImmuCellAI algorithm. **(A)** The relative abundances of γδ T cells among the γδT-hi, γδT-lo and normal groups. **(B)** The relative abundances of CD4+ T cells and their subsets among the γδT-hi, γδT-lo and normal groups. **(C)** The relative abundances of CD8+ T cells and their subsets among the γδT-hi, γδT-lo and normal groups. **(D)** The relative proportions of B cells, NKT cells, MAIT, NK cells, dendritic cells, monocytes, macrophages and neutrophils among the γδT-hi, γδT-lo and normal groups. *P*-values were calculated by student's *t*-test. CD4 naïve, naïve CD4+ T cells; Th1, T helper cells type 1; Th2, T helper cells type 2; Th17, T helper cells type 17; Tfh, follicular helper T cells; iTreg, induced regulatory T cells; nTreg, natural regulatory T cells; Tr1, type 1 regulatory T cellss; CD8 naïve, naïve CD8+ T cell; Tcm, central memory T cells; Tem, effector memory T cells; Tc, cytotoxic T cells; Tex, exhausted T cells; NKT cells, natural killer T cells; MAIT, mucosal-associated invariant T cells; NK cells, natural killer cells. ns, not significant; *P < 0.05; **P < 0.01; ***P < 0.001; ****P < 0.0001.

Since the γδ T cell abundance was positively correlated with improved survival of HNSCC patients, we next tried to explore the potential reasons for these new findings. We used the new online immune cell proportion estimation tool ImmuCellAI to analyze the abundance of 24 kinds of immune cells in the RNA-seq results of 537 samples. This tool was reported to possess a high accuracy in predicting the composition of immune cells in tumor tissues, especially T cells (30). The results showed that the γδ T cell proportion in the γδT-hi group was significantly increased (*P* < 0.0001, Figure 2A), which was consistent with our grouping method. Moreover, both CD4+ T cell and CD8+ T cell abundances in the γδT-hi group increased significantly (*P* < 0.0001, Figures 2B,C). Among CD4+ T cells, T helper cells type 1 (Th1), T helper cells type 2(Th2), and follicular helper T cells (Tfh) were significantly increased in the γδT-hi group (*P* < 0.0001, Figure 2B), while the proportion of naïve CD4+ T cells and T helper cell type 17 (Th17) was lower than those in γδT-lo or normal groups (Figure 2B). However, the increase in CD4+ T cell infiltration was also accompanied by the increase of regulatory T cell abundance in the γδT-hi group (Figure 2B). In CD8+ T cells, central memory T cells, cytotoxic T cells, and exhausted T cells accumulated in the γδT-hi group, while the proportion of naïve CD8+ T cells decreased significantly, but the proportion of effector memory T cells was not increased when compared to the γδT-lo or normal groups (Figure 2C). In addition, the other cell abundances showed that the relative abundances of B cells, natural killer (NK) cells and macrophages increased significantly in the γδT-hi group, and the proportion of natural killer T (NKT) cells, monocytes and neutrophils decreased significantly (Figure 2D). To verify these results, we used another online immune cell fraction estimation method, CIBERSORTx, to calculate the relative abundance of immune cells in the HNSCC samples. The results, consistent with those of ImmuCellAI algorithm, showed that the CD8+ T cells, activated CD4+ T cells, Tfh and NK cells in the γδT-hi group were significantly increased (Supplementary Figure 2A), while the monocytes and neutrophils were decreased (Supplementary Figure 2B). It has been reported that tumor-infiltratingTh17, neutrophils, and monocytes are able to promote tumor progression (32–34), and thus the relatively low abundance of Th17 cells, neutrophils and monocytes also contributed to the better OS in the γδT-hi group. Furthermore, M1 macrophages with an antitumor effect were increased in the TME of the γδT-hi group, while the proportion of M2 macrophages, which promote tumor development, was significantly reduced (Supplementary Figure 2B). Furthermore, we have found a similar immune cell distribution pattern between the γδT-hi and γδT-lo groups in the CESC dataset calculated by ImmuCellAI algorithm (Supplementary Figures 1C–F), indicating the immune cell infiltration may be affected by γδ T cells in the TME across different types of cancer.

### Genes Related to T Cell Effector Functions and Chemokine Production Are Highly Expressed in the γδT-hi Group

**Figure 3:**
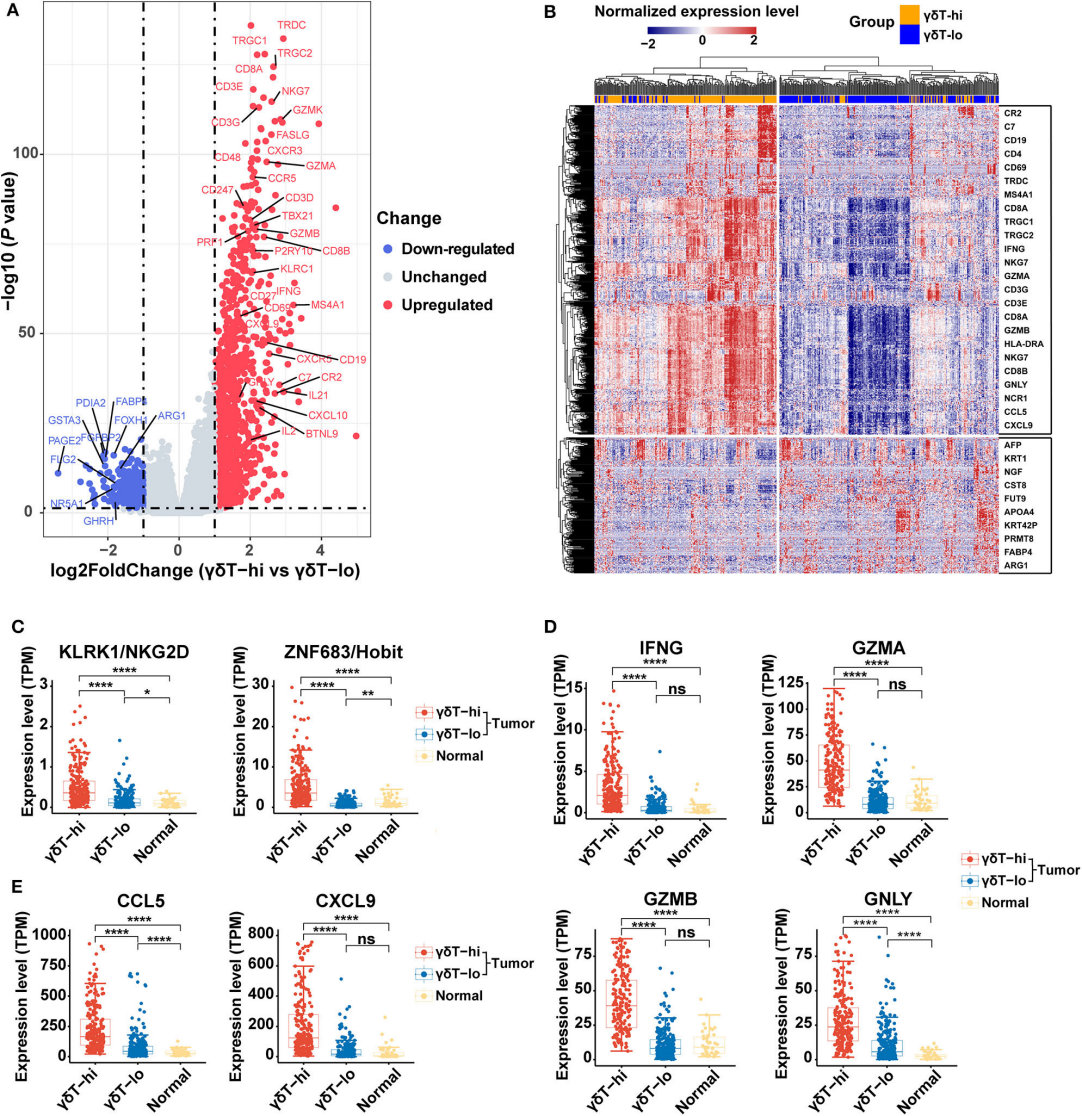
Differentially expressed genes among the γδT-hi, γδT-lo and normal groups in HNSCC using R package DESeq2. **(A)** Volcano plot of log_2_ (Fold Change) vs. –log_10_ (*P-*value) shows the differentially expressed genes between the γδT-hi and γδT-lo groups. Red points represent the significantly upregulated genes in the γδT-hi group, blue points represent the significantly down-regulated genes in the γδT-hi group (upregulated in γδT-lo group), and gray points represent the genes without significant differences (unchanged). Dotted lines show a *P-*value of 0.05 (horizontal) and a fold change of ±2 (vertical). **(B)** Cluster heatmap showing the differential expressed genes between the γδT-hi (orange) and γδT-lo (light blue) groups. All of the expression values of each gene were rescaled to the range from −2 to 2 by Z-Score normalization. The red color in the heatmap indicates the genes that are upregulated, blue color indicates the genes that are down-regulated. Some representative genes were labeled in the boxes on the right side, the upper box contains some representative genes that are upregulated in the γδT-hi group, whereas the lower box contains some representative genes that are down-regulated in the γδT-hi group. **(C)** NKG2D (KLRK1) and Hobit (ZNF683) expression levels among the γδT-hi, γδT-lo and normal groups. **(D)** IFNG/GZMA/GZMB/GNLY expression levels among the three groups. **(E)** Expression levels of chemokines CCL5/CXCL9 among the three groups. Each dot represents the expression value (TPM) of the specific gene in each sample. *P*-values were calculated by student's *t*-test. ns, not significant; *P < 0.05; **P < 0.01; ****P < 0.0001.

We further analyzed the differential genes between γδT-hi and γδT-lo groups by using R package DESeq2. As shown in the Figures 3A,B, a total of 1,113 genes were upregulatedand 329 genes were down-regulated in the γδT-hi group (|log2FoldChange| > 1, Padj <0.05, Supplementary Table 1). Among these DEGs, genes encoding CD3 (CD3E/CD3G/CD3D), and genes related to Th1 cells (CD4/TBX21), CD8+ T cells (CD8A/CD8B), B cells (CD19/MS4A1), and NK cells (NCR1/NKG7) were significantly upregulated (Figure 3A), which was in line with the increased proportion of Th1/CD8+ T cells/B cells/NK cells in the γδT-hi group. In contrast, the gene KRT1 related to keratinocytes, the tumor marker AFP (alpha-fetoprotein), and ARG1 (encoding arginae1) related to inhibiting CD8+ T cell function were upregulated in the γδT-lo group (Figure 3B). NKG2D, the surface marker of activated cytotoxic γδ T cells, and Hobit, the transcription factor that enhance the cytotoxicity of γδ T cells (35), were both upregulated in the γδT-hi group compared to those in the γδT-lo or normal group (Figure 3C). At the same time, the expression level of cytokines and granzymes (IFNG/GZMA/GZMB/GNLY) were also significantly increased in the γδT-hi group (Figure 3D). These results revealed the enhanced effector functions of tumor-infiltrating T cells of the γδT-hi group. Previous studies have reported that γδ T cells could release chemokines CCL5 and CXCL9 to attract other T cells to fight against pathogens or tumors (26, 36). In the γδT-hi group, we found that these two chemokines were also significantly upregulated, and they are reported to be essential for the recruitment of CD8+ T cells to the TME for tumor cell killing (Figure 3E). Therefore, γδ T cells may also recruit CD8+ T cells via chemokine release.

### Gene Sets and Cell Pathways Related to T Cell Activation, Proliferation, Chemokine Production and Cytotoxicity Are Enriched in the γδT-hi Group

**Figure 4:**
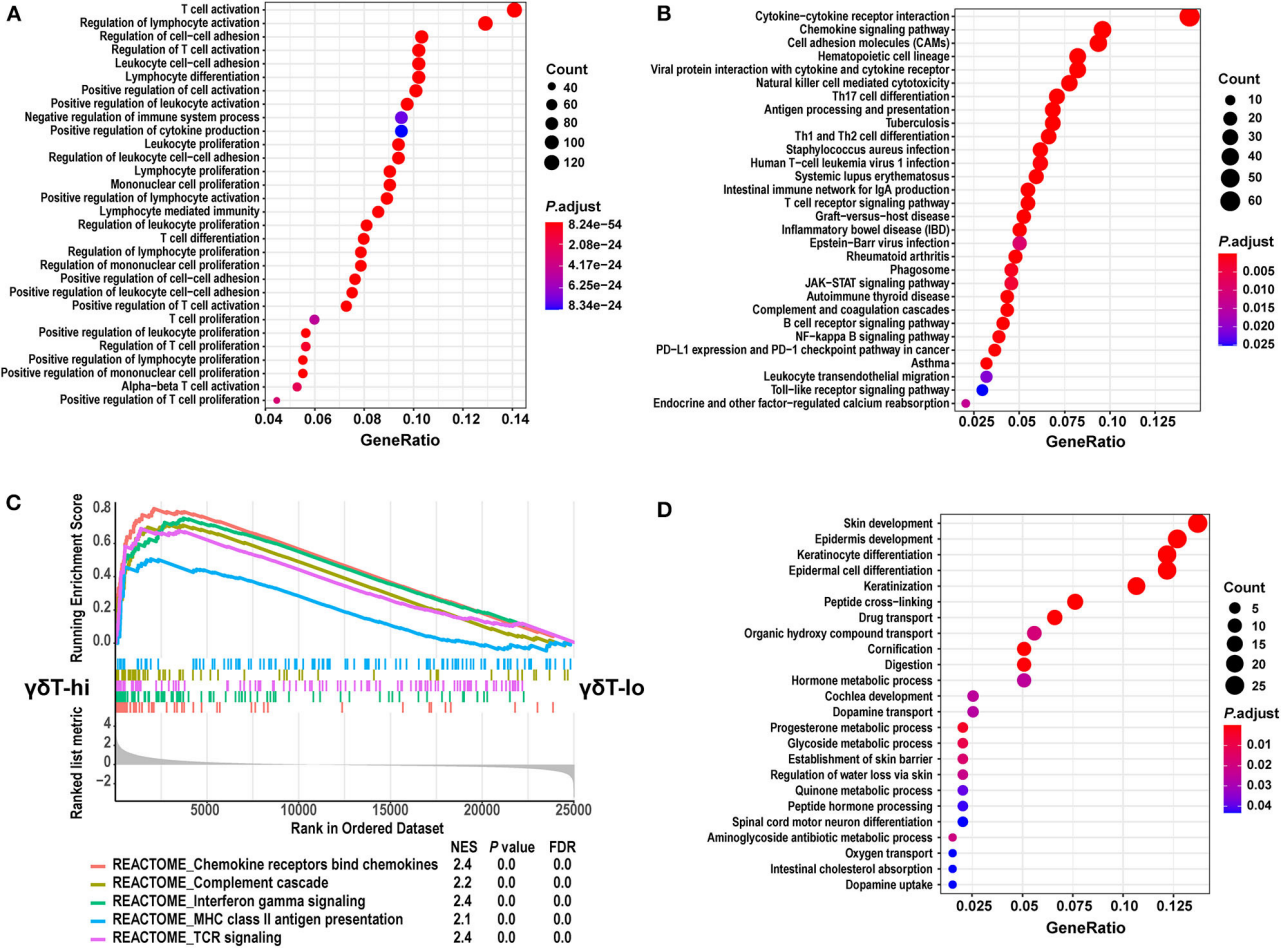
GO and KEGG term enrichment analyses for the differentially expressed genes and GSEA analysis between the γδT-hi and γδT-lo groups. **(A)** The top 30 enrichment results from GO biological process terms in γδT-hi group. **(B)** The top 30 enrichment results from KEGG pathway terms in the γδT-hi group. **(C)** GSEA analysis shows the 5 REACTOME pathways that are enriched in the γδT-hi group. **(D)** The enrichment results from GO biological process terms in the γδT-lo group. GO and KEGG enrichment analyses were calculated by R package clusterProfiler (version: 3.16.1), the terms were not mutual excluded, adjusted *P*-values (P. adjust) were calculated by hypergeometric distribution. The size of dots indicates the count of significantly upregulated genes in γδT-hi or γδT-lo group that overlap with the specific geneset in GO or KEGG database, the color of dots indicates the adjusted *P*-values, red color indicates the smaller adjusted *P*-values. GeneRatio means the relative proportion of the overlapped genes in the specific dataset in GO or KEGG database. GSEA analysis was performed using the REACTOME database in MSigDB (version 7.1), and *P*-values for GSEA analyses were calculated by permutation tests. The lower part of **(C)** shows the genes that are ranked from the most upregulated in γδT-hi group (left side) to the most upregulated in γδT-lo group (right side). The middle part shows the distribution of genes in specific dataset in the ranked list. The upper part shows the running enrichment score of each dataset. NES, normalized enrichment score; FDR, false discovery rate.

We then performed GO and KEGG term enrichment on the DEGs in the γδT-hi group and the γδT-lo group, respectively. The results showed that the genes upregulated in the γδT-hi group enriched the functions related to T cell activation, proliferation, differentiation, cytokine production, and cell-cell adhesion in the GO biological process (Figure 4A). In the KEGG results, the γδT-hi group was enriched for terms including cytokine-cytokine receptor interaction, chemokine signaling pathway, T cell and B cell receptor pathway, Th1 and Th2 cell differentiation, and others (Figure 4B). Furthermore, it was noted that chemokine bind chemokine receptors, complement cascade, interferon-γ pathway, and MHC-II antigen presentation were significantly enriched in the γδT-hi group through the GSEA analysis (*P* < 0.05, FDR <0.25, Figure 4C). The genes that participate in the complement pathway and antigen presentation, including C7/CR2/HLA-DRA, were also found in the γδT-hi group (Figures 3A,B), indicating that the complement cascade and antigen presentation were also involved in the γδ T cell-mediated immune response. In contrast, only biological processes such as cell keratinization and hormone metabolism were enriched in the γδT-lo group (Figure 4D). It was reported that γδ T cells could also serve as antigen-presenting cells to activate conventional T cells in infectious disease or the TME (37), our results, consistent with these previous studies, showed that γδ T cells may exert antitumor responses by activating and recruiting effector T cells or NK cells to the TME and through MHC-II antigen presentation, but the exact mechanism is still unknown.

### The Abundance of γδ T Cells Is Positively Associated With the Expression of Butyrophilin Family Proteins in Tumor Cells

**Figure 5:**
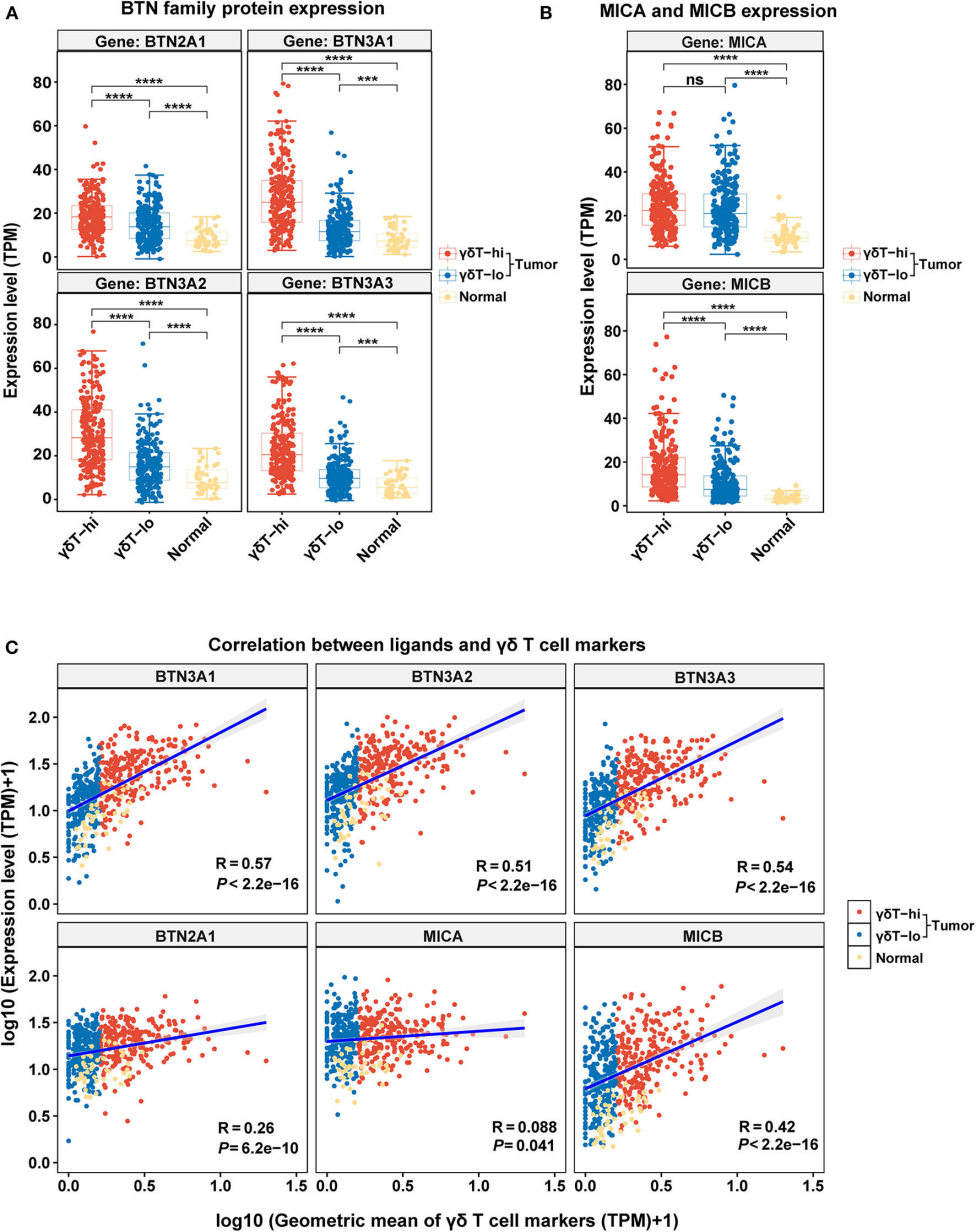
The boxplots showing the expression levels of γδ T cell ligands among the γδT-hi, γδT-lo and normal groups and the correlation analysis between the γδT markers and ligands. **(A)** BTN2A1/BTN3A1/BTN3A2/BTN3A3 expression levels among the γδT-hi, γδT-lo and normal groups. **(B)** MICA/MICB expression levels among the γδT-hi, γδT-lo and normal groups. Each point represents the expression value (TPM) of the specific gene in each sample. *P*-values were calculated by student's *t*-test in **(A,B)**. **(C)** Linear regression analyses show the correlation between the ligands and the geometric mean of the γδT markers. For the linear regression analysis, all the expression data were transformed into log_10_ (TPM+1). Each point represents the expression value [log_10_ (TPM + 1)] of the specific gene in each sample. *R*-values and *P*-values were calculated by linear regression analyses. ns, not significant; ***P < 0.001; ****P < 0.0001.

We have explored the possible antitumor mechanism of γδ T cells in HNSCC, but the factors that contribute to the increase in γδ T cells in the TME are still unclear. Thus, we explored the expression levels of BTNs and NKG2DLs among the three groups and performed the linear correlation analysis between the expression levels of BTNs or NKG2DLs and the γδ T cell markers. We found that the expression levels of BTN2A1/BTN3A1/BTN3A2/BTN3A3 in the γδT-hi group were significantly higher than those in the γδT-lo group or normal group (*P* < 0.0001), and the expression levels of BTNs in the normal control group were the lowest (Figure 5A). Through linear regression analysis, we found that there was a relatively strong correlation between BTN3A1/BTN3A2/BTN3A3 and the γδ T cell markers, while the correlation between BTN2A1 and the γδ T cell markers was relatively weak (Figure 5C). Furthermore, the results showed that only MICB expression in the γδT-hi group was significantly higher than that in the γδT-lo or normal groups, while MICA expression was not significantly higher than that in the γδT-lo group (Figure 5B). Linear regression analysis showed that MICB expression had moderate correlation with γδ T cell markers, while MICA showed nearly no correlation (Figure 5C). In contrast, the expression of ULBP family proteins in γδT-hi was lower than that in the γδT-lo group, suggesting that activation of the γδ T cells in HNSCC might not be related to the ULBP family proteins (Supplementary Figure 3A). However, when we dichotomized the HNSCC cohort based on the median expression levels of BTN3A1/BTN3A2/BTN3A3/BTN2A1, we found that although there was no statistical significance between the high and low groups, the patients with higher expression levels of the BTN family proteins showed better overall survival than patients whose expression levels were lower than the median expression levels (*P* > 0.05, Supplementary Figure 3B).

## Discussion

In the present study, we used TCGA dataset to demonstrate that the high abundance of γδ T cells in HNSCC was positively associated with an improved prognosis of patients, possibly due to the enhanced antitumor effect of γδ T cells and the recruitment of CD8+ T cells to the TME. Finally, we found that the increased γδ T cell abundance in the TME was associated with upregulation of BTN family proteins and the NKG2D ligand MICB in tumor cells, indicating that the activation of γδ T cells may be associated with BTN family proteins and MICB.

Tumor-infiltrating lymphocytes play a vital role in the control of tumor progression, with different clinical effects (38). Similar to αβ T cells, γδ T cells are also found in various types of human tumors. Both Vδ1 T and Vδ2 T cells have been found in various epithelial tumors, such as lung cancer, renal cancer, melanoma, and colorectal cancer (12). By applying the CIBERSORT analysis in a pan-cancer analysis of over 18,000 human tumors in TCGA data, γδ T cells were found to be the most favorable prognostic populations among all types of tumor-infiltrating leukocytes across all types of tumors (39). However, whether γδ T cells exert antitumor or protumor effects in the TME has not been validated. Vδ2 T cells activated by non-peptide phosphoantigens *in vitro* have been proven to inhibit tumor growth both *in vitro* and *in vivo* (40, 41). Currently, the results of phase I clinical trials on advanced lung cancer, renal cancer and melanoma have revealed that adoptive Vδ2 T cell transfer therapies have shown certain antitumor effects (42). However, other studies have reported that Vδ2 T cells can also promote tumor progression (43). Under specific stimulation, Vδ2 T cells can be differentiated into subsets endowed with Th17 or Treg characteristics, exerting protumor and immunosuppressive effects by producing IL-17 and IL-10, respectively (44). In addition, Vδ1 T cells have also been found to have dual effects in tumor immunity. In patients with hepatocellular carcinoma (45) and gastric cancer (46), a high infiltration of Vδ1 T cells is associated with a longer survival time, but in patients with breast cancer (47) or colorectal cancer (13), high infiltration of γδT cells is associated with poor prognosis. Our results have showed that patients with higher γδ T cell proportions were correlated with lower T stages, and a longer overall survival of HNSCC patients both in univariate and multivariate analyses, suggesting that γδ T cells may be involved in the antitumor immunity in HNSCC. However, a previous study has shown that there was no correlation between the proportions of γδ T cells and tumor stages in HNSCC patients (14), which is seemingly paradoxical with our results. The possible explanation is that the previous study only compared the γδ T cell abundance in peripheral blood, whereas γδ T cells in the TME were not analyzed. The composition of lymphocytes in the peripheral blood and the TME may be different, and due to the lack of RNA-seq data from the peripheral blood in HNSCC patients of TCGA dataset, it is not possible to explore whether γδ T cells in peripheral blood reflect those in the TME.

γδ T cells are considered as a bridge between innate and adaptive immune responses. Apart from direct tumor cell killing, γδ T cells interact with other innate and adaptive immune cells in the TME to exert indirect antitumor responses. IFN-γ secreted by γδ T cells promotes the upregulation of MHC-I molecules on tumor cells and positively regulates the antitumor function of CD8+ T cells (48). In addition, γδ T cells activate NK cells through the CD137/CD137L axis, promoting cytotoxicity in solid tumors (49). In the presence of P-Ags and interleukin 21, Vδ2 T cells can also express Tfh cell-related markers, such as ICOS, CD40L and CXCR5 and these Tfh-like γδ T cells (γδ Tfh) secrete cytokines, such as IL-4 and CXCL13, to increase antibody production of B cells (48). In addition, γδ T cells also serve as antigen presenting cells to activate CD8+ T cells and Th1 cells (37). Consistent with the previous research, our study found that the high abundance of γδ T cells was accompanied by the increase in CD8+ T cells, Th1 cells, Tfh cells, B cells, and other cell subsets. Moreover, through gene ontology and pathway enrichment analyses, it was indicated that γδ T cells might be involved in the activation of the IFN-γ signaling pathway, antigen presentation, chemokine secretion and other biological processes.

The unexpected discoveries in our study are that regulatory T cells, which are known as negative regulators of the immune response and contribute to tumor progression, accumulated in the γδT-hi group. Previous studies have revealed that higher Treg abundance was associated with shorter overall survival in renal cancer, breast cancer or melanoma (50), while high infiltration of Foxp3+ Treg cells in HNSCC was associated with an improved overall survival (51), and studies have revealed that higher CD8+ T cell or NK cell infiltration was usually accompanied by higher infiltration of Tregs in HNSCC (51); therefore it is hypothesized that Tregs are trafficked into the TME after CD8+ T and NK cells as a negative feedback to prevent the excessive inflammation mediated by CD8+ T cells and NK cells. In addition, we also discovered that the proportion of exhausted CD8+ T cells accumulated but that NKT cells were decreased in the γδT-hi group, which was seemingly paradoxical with our results. Although it is known that T cell exhaustion is a state in which antigen-specific CD8+ T cells undergo a progressive and hierarchical loss of effector functions during chronic antigen stimulation in the tumor microenvironment (52), this process does not occur in other bystander CD8+ T cells (53), and recent studies have also revealed that it is the exhausted T cells that exert the antitumor effect in an antigen-specific manner (53); previous studies have also discovered this phenomenon that HNSCC patients with higher expression of exhausted markers, including PDCD1, TIM3, and CD39, were associated with a better OS (54). Therefore, the more antigen-specific T cells that enter the TME, the greater chance they may terminally differentiate into exhausted T cells. For NKT cell, although it is an innate-like lymphocyte with invariant TCR that exerts potent antitumor immunity (55), the activation and expansion of NKT cells requires the presentation of α-galactosylceramide (α-GalCer) (56), a specific glycolipid antigen that is almost absent in the TME, and thus the expansion capacity of NKT cells in the TME is restrained. Therefore, compared to other T cell subsets that proliferate rapidly in the TME in the γδT-hi group, including CD8+ T cells, CD4+ T cells or γδ T cells, the number of NKT cells remains unchanged, resulting in the relatively lower proportions compared to the γδT-lo group.

As a novel group of type I transmembrane proteins of the immunoglobulin superfamily, the butyrophilin family of proteins shares a high homology with the B7 family proteins of the extracellular domains, suggesting that BTNs may also possess immunoregulatory functions (57). Among the BTN families, BTN1A1 and BTN2A2 have been reported to inhibit the activation and immune response of conventional T cells (58, 59). However, BTN3A1, along with BTN3A2 and BTN3A3, was required for P-Ag presentation and activation of Vδ2 T cells (18, 19). In addition, recent studies have revealed that BTN2A1 was another ligand that cooperated with BTN3A1 to activate Vδ2 T cells (20, 60).Studies have shown that a TP53 gene mutation would lead to the activation of the mevalonate pathway in cancer cells, resulting in the accumulation of isopentenyl pyrophosphate (IPP) and its isomer dimethylallyl pyrophosphate (DMAPP) in tumor cells (61). These metabolites would be taken up by BTNs and presented to Vδ2 T cells, leading to the activation of γδ T cells and an enhanced antitumor immune response (62, 63). Studies have shown that through binding to P-Ags presented by BTN3A1 and BTN2A1 on infected or malignant cells, γδ T cells could be activated, proliferate rapidly, and induce anti-infection or antitumor responses through IFN-γ production (20). Notably, it has been demonstrated that the B30.2 domain of BTN3As can bind P-Ag and drive the activation of Vδ2 T cells through conformational changes of the extracellular domains (64, 65), and periplakin and RhoB are the key proteins that play important roles in spatial rearrangement of BTN3As following intracellular P-Ag sensing (66–68). But how then do P-Ag enter the cells to initiate γδ T cell activation following binding to cytosolic B30.2 is needed to be clarified. Recent studies have found that a higher expression of BTN3A2 in ovarian cancer or triple negative breast cancer is positively correlated with an increased T cell infiltration and a better prognosis (23, 69). Our results, in line with previous studies, showed that γδ T cells in the TME might be activated in a butyrophilin-dependent manner, and mediated an antitumor response against HNSCC.

Apart from the interaction of the BTN family proteins and TCRγδ, the binding of NKG2D with NKG2DLs is the costimulatory signaling pathway that activates γδ T cells. It was reported that tumor cells expressing NKG2DLs (both MIC proteins and ULBP proteins) were more susceptible to γδ T cell-mediated lysis (70). However, although the expression level of NKG2D was much higher in the γδT-hi group, only MICB expression was upregulated in the γδT-hi group among the NKG2DLs, while MICA expression was not upregulated, with the ULBP family proteins showing the opposite results. The possible explanation for these results is that the activation of γδ T cells is not primarily through the NKG2DL-NKG2D pathway, and another possible explanation is that apart from the NKG2DLs expressed on tumor cells, the soluble NKG2DLs can also suppress the antitumor response of γδ T cells (71). The exact mechanisms of how NKG2DLs activate γδ T cells in head and neck cancer still need further exploration. Collectively, our results showed that γδ T cells might be activated and exert antitumor effects mainly through the recognition of BTN family proteins, which might be promising targets for γδ T-cell mediated immunotherapy.

In conclusion, our results showed that the abundance of γδ T cells in the tumors was positively associated with the improvement of prognosis in HNSCC patients. This antitumor effect might be attributed to the enhancement of γδ T cell-mediated cytotoxicity and the recruitment and activation of other antitumor lymphocytes. BTN2A1 and BTN3As might be the direct ligands that activate γδ T cells in head and neck cancers. Our results provide a new perspective of the HNSCC microenvironment, and provide potential targets for immunotherapy of HNSCC, which deserve further exploration.

## Data Availability Statement

All datasets generated for this study are included in the article/Supplementary Material.

## Author Contributions

ZW contributed to conception and design of the study. HL contributed to the design of the study and acquisition and analysis of the data. WD, JG, and DW contributed to the analysis and interpretation of the data. SW, LY, DL, WX, LW, and JF contributed to the interpretation of the data. HL, WD, and JG wrote the first draft of the manuscript. DW, SW, LY, DL, and WX wrote sections of the manuscript. All authors contributed to manuscript revision, read, and approved the submitted version.

## Conflict of Interest

The authors declare that the research was conducted in the absence of any commercial or financial relationships that could be construed as a potential conflict of interest.

## Supplementary Material

Supplementary Figure 1 The distribution of T stages and overall survival curves between γδT-hi (*n* = 139) and γδT-lo (*n* = 139) groups and the immune cell abundances among the γδT-hi, γδT-lo and normal groups (*n* = 3) in the CESC dataset. **(A)** Five-year overall survival curves between the γδT-hi and γδT-lo groups. **(B)** The proportions of patients in each T stage (1–4) in the γδT-hi and γδT-lo groups. **(C)** The relative abundances of γδ T cells among the γδT-hi, γδT-lo and normal groups calculated by ImmuCellAI algorithm. **(D)** The relative abundances of CD4+ T cells and their subsets among the γδT-hi, γδT-lo and normal groups calculated by ImmuCellAI algorithm. **(E)** The relative abundances of CD8+ T cells and their subsets among the γδT-hi, γδT-lo and normal groups calculated by ImmuCellAI algorithm. **(F)** The relative abundances of B cells, NKT cells, MAIT, NK cells, dendritic cells, monocytes, macrophages and neutrophils among the γδT-hi, γδT-lo and normal groups calculated by ImmuCellAI algorithm. *P*-values were calculated by student's *t*-test. CD4 naïve, naïve CD4+ T cells; Th1, T helper cells type 1; Th2, T helper cells type 2; Th17, T helper cells type 17; Tfh, follicular helper T cells; iTreg, induced regulatory T cells; nTreg, natural regulatory T cells; Tr1, type 1 regulatory T cellss; CD8 naïve, naïve CD8+ T cell; Tcm, central memory T cells; Tem, effector memory T cells; Tc, cytotoxic T cells; Tex, exhausted T cells; NKT cells, natural killer T cells; MAIT, mucosal-associated invariant T cells; NK cells, natural killer cells. ns, not significant; ^*^*P* < 0.05; ^**^*P* < 0.01; ^***^*P* < 0.001; ^****^*P* < 0.0001.

Supplementary Figure 2 Relative abundance of cell types among the γδT-hi, γδT-lo and normal groups in HNSCC by using CIBERSORTx. **(A)** The proportions of lymphocyte subsets (B cells, T cells and NK cells) among the γδT-hi, γδT-lo and normal groups. **(B)** The relative proportions of myeloid cell subsets (monocytes, macrophages dendritic cells, mast cells, eosinophils and neutrophils) among the γδT-hi, γδT-lo and normal groups. *P*-values were calculated by student's *t*-test. ns, not significant; ^**^*P* < 0.01; ^***^*P* < 0.001; ^****^*P* < 0.0001.

Supplementary Figure 3 The expression levels of ULBP family proteins among the γδT-hi, γδT-lo and normal groups, and the association between BTN family proteins and 5-year OS. **(A)** The expression levels of ULBP1/ULBP2/ULBP3/RAET1E/RAET1G/RAET1L among the γδT-hi, γδT-lo and normal groups. Each point represents the expression value (TPM) of the specific gene in each sample. *P* values were calculated by student's *t*-test. **(B)** HNSCC patients were dichotomized into high and low group based on the median expression of BTN3A1/BTN3A2/BTN3A3/BTN2A1, and the OS curves were drawn for the high and low group, respectively. ns, not significant; ^**^*P* < 0.01; ^***^*P* < 0.001; ^****^*P* < 0.0001.
