# Supplementary figures and images for: High Abundance of Intratumoral γδ T Cells Favors a Better Prognosis in Head and Neck Squamous Cell Carcinoma: A Bioinformatic Analysis
Source: Front Immunol. 2020 Sep 30;11:573920. doi: 10.3389/fimmu.2020.573920 (PMC7555127; doi:10.3389/fimmu.2020.573920)

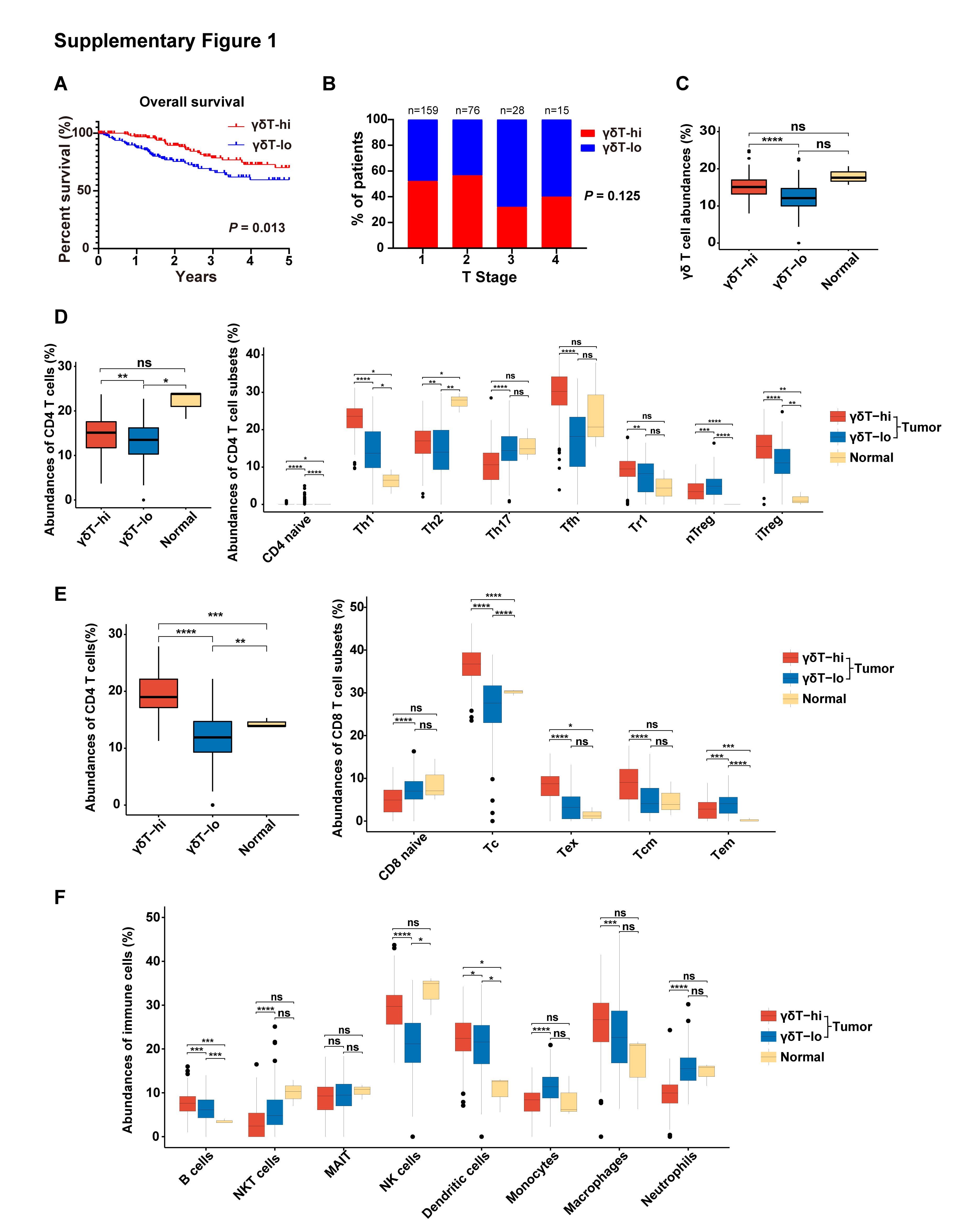

Supplement: Supplementary Figure 1 — The distribution of T stages and overall survival curves between γδT-hi (n = 139) and γδT-lo (n = 139) groups and the immune cell abundances among the γδT-hi, γδT-lo and normal groups (n = 3) in the CESC dataset. (A) Five-year overall survival curves between the γδT-hi and γδT-lo groups. (B) The proportions of patients in each T stage (1–4) in the γδT-hi and γδT-lo groups. (C) The relative abundances of γδ T cells among the γδT-hi, γδT-lo and normal groups calculated by ImmuCellAI algorithm. (D) The relative abundances of CD4+ T cells and their subsets among the γδT-hi, γδT-lo and normal groups calculated by ImmuCellAI algorithm. (E) The relative abundances of CD8+ T cells and their subsets among the γδT-hi, γδT-lo and normal groups calculated by ImmuCellAI algorithm. (F) The relative abundances of B cells, NKT cells, MAIT, NK cells, dendritic cells, monocytes, macrophages and neutrophils among the γδT-hi, γδT-lo and normal groups calculated by ImmuCellAI algorithm. P-values were calculated by student's t-test. CD4 naïve, naïve CD4+ T cells; Th1, T helper cells type 1; Th2, T helper cells type 2; Th17, T helper cells type 17; Tfh, follicular helper T cells; iTreg, induced regulatory T cells; nTreg, natural regulatory T cells; Tr1, type 1 regulatory T cellss; CD8 naïve, naïve CD8+ T cell; Tcm, central memory T cells; Tem, effector memory T cells; Tc, cytotoxic T cells; Tex, exhausted T cells; NKT cells, natural killer T cells; MAIT, mucosal-associated invariant T cells; NK cells, natural killer cells. ns, not significant; *P < 0.05; **P < 0.01; ***P < 0.001; ****P < 0.0001. [file Image_1.TIF]

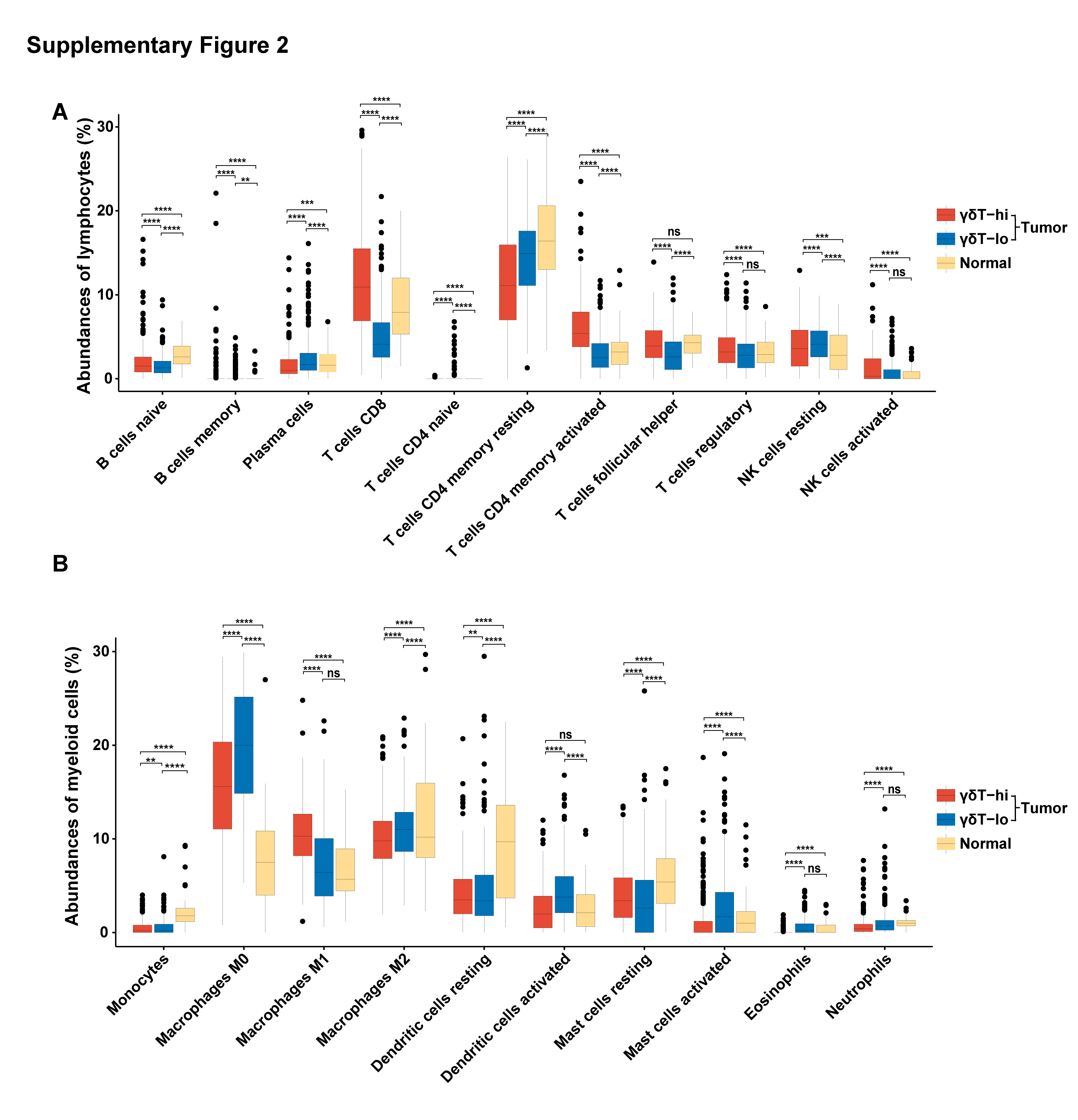

Supplement: Supplementary Figure 2 — Relative abundance of cell types among the γδT-hi, γδT-lo and normal groups in HNSCC by using CIBERSORTx. (A) The proportions of lymphocyte subsets (B cells, T cells and NK cells) among the γδT-hi, γδT-lo and normal groups. (B) The relative proportions of myeloid cell subsets (monocytes, macrophages dendritic cells, mast cells, eosinophils and neutrophils) among the γδT-hi, γδT-lo and normal groups. P-values were calculated by student's t-test. ns, not significant; **P < 0.01; ***P < 0.001; ****P < 0.0001. [file Image_2.TIF]

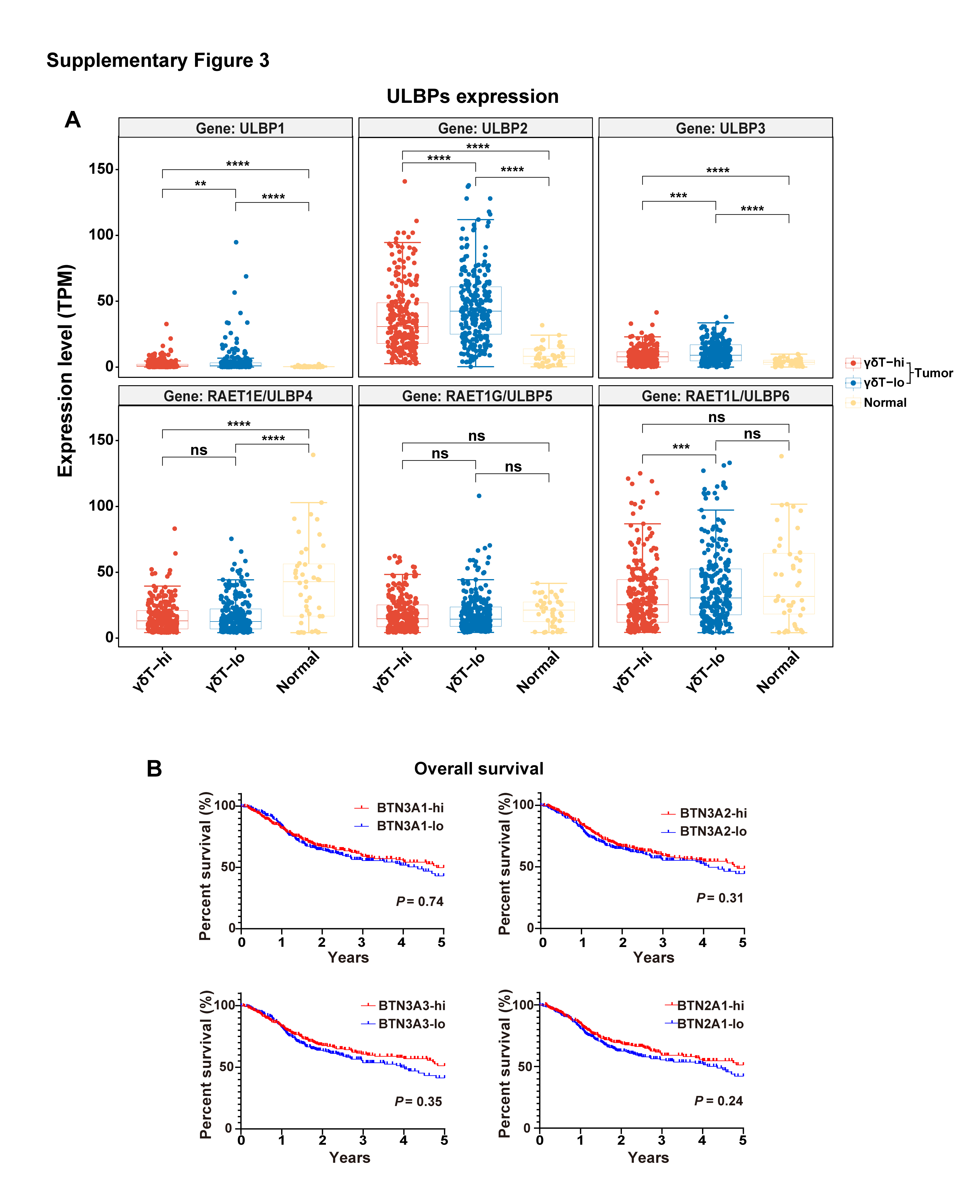

Supplement: Supplementary Figure 3 — The expression levels of ULBP family proteins among the γδT-hi, γδT-lo and normal groups, and the association between BTN family proteins and 5-year OS. (A) The expression levels of ULBP1/ULBP2/ULBP3/RAET1E/RAET1G/RAET1L among the γδT-hi, γδT-lo and normal groups. Each point represents the expression value (TPM) of the specific gene in each sample. P values were calculated by student's t-test. (B) HNSCC patients were dichotomized into high and low group based on the median expression of BTN3A1/BTN3A2/BTN3A3/BTN2A1, and the OS curves were drawn for the high and low group, respectively. ns, not significant; **P < 0.01; ***P < 0.001; ****P < 0.0001. [file Image_3.TIF]
